# Stigma Resistance and Its Associated Factors among People with Bipolar Disorder at Amanuel Mental Specialized Hospital, Addis Ababa, Ethiopia

**DOI:** 10.1155/2020/7917965

**Published:** 2020-09-26

**Authors:** Nigus Alemnew Engidaw, Eyosiyas Yeshialem Asefa, Zelalem Belayneh, Abate Dargie Wubetu

**Affiliations:** ^1^College of Health Sciences, Debre Berhan University, Debre Berhan, Ethiopia; ^2^Department of Psychiatry, College of Health and Medical Science, Dilla University, Dilla, Ethiopia

## Abstract

**Background:**

Stigma resistance is the capacity to cope and remain unaffected by mental illness stigmatization. In bipolar patients, having low stigma resistance may result in a higher internalized stigma, low self-esteem, and poor treatment outcome. In Ethiopia, the prevalence of stigma resistance among bipolar patients is not well known. Therefore, this study is aimed at assessing the prevalence of stigma resistance and its associated factors among bipolar patients at Amanuel Mental Specialized Hospital, Addis Ababa, Ethiopia.

**Method:**

An institutional-based cross-sectional study was conducted from May 8^th^ to June 14^th^, 2016, at Amanuel Mental Specialized Hospital. The study participants were selected using a systematic random sampling technique. The stigma resistance subscale of the internalized stigma of mental illness was used to measure stigma resistance. Bivariable and multivariable logistic regression was computed to identify factors associated with stigma resistance. Accordingly, variables with *P* values of less than 0.05 were considered as statistically significant predictors of stigma resistance with a 95% confidence interval.

**Results:**

In this study, 418 participants completed the interview with a response rate of 98.8%. The prevalence of low stigma resistance was 56.9% (95%CI = 51.9‐61.6%). Being unemployed (AOR = 1.65; 95%CI = 1.35‐1.87), high internalized stigma (AOR = 3.04; 95%CI = 1.83‐5.05) and low self-esteem (AOR = 2.13; 95%CI = 1.72‐6.76) were significantly associated with low stigma resistance. *Conclusions and Recommendation*. More than half of the bipolar patients attending the Amanuel Mental Specialized Hospital had low stigma resistance. Therefore, stigma reduction programs have focused on improving self-esteem and reducing internalized stigma to increase their stigma resistance. Mental health information dissemination regarding community support and reengagement of people with bipolar disorder is highly recommended.

## 1. Background

Bipolar disorder (BD) is one of the most common, severe, disabling, and chronic mental disorders. It is manifested by manic, hypomanic, or major depressive episodes [1, 2]. According to the World Health Organization (WHO) report, the global burden of disease and lifetime risk of BD are 2.6% and 7.8%, respectively [3, 4]. BD has an enormous and devastating social, economic, and health impact on sufferers and their families [5, 6]. Since this disorder is chronic, severe, and episodic by its nature, it has been shown to be related with unemployment, low productivity, poor social functioning, poor quality of life, disability, marital failure, high treatment costs, significant family burden, and poor quality of life [7–9]. Because of its relapsing nature, disability, and negative attitude of the society towards mental disorders, BD is also associated with stigma [10, 11].

Stigma is a multidimensional construct that involves feelings, attitudes, and behaviors. It encompasses three main constituents: negative stereotypes, prejudice, and discrimination [12]. Stigma is also a common and frequent burden of individuals with severe psychiatric disorders, their families, and caregivers [13–17]. The reactions to stigma by people with mental illness vary. Some people become energized and empowered (stigma resistance), few remain indifferent and unaffected, while others internalize stigma [18]. Internalized stigma is the process of applying negative stereotypes and stigmatizing attitudes to oneself and may exhibit behaviors like secrecy and withdrawal to cope with this discrimination [19]. If there is a high internalized stigma, stigma resistance (SR) will be low. Developing matured coping strategies such as psychological resilience and having better stigma resistance abilities are very important to tackle internalized stigma [20, 21].

Resilience has been conceptualized as “the capacity of a dynamic system to withstand or recover from significant challenges that threaten its stability, viability or development” [22]. Accordingly, resilience has been suggested to be relevant for coping with mental illness [23]. A previous study has shown that there is a positive correlation between stigma resistance and resilience [24]. SR is the capacity to resist, counteract, or remain unaffected by mental illness stigmatization [25]. It is a continuous and active process of using one's experiences, skills, and knowledge to develop an optimistic identity toward mental illness. Theoretical work on SR suggests that resisting stigma has an important role in fighting internalized stigma, facilitating the recovery from psychiatric illness, adherence, promoting their self-esteem, empowerment, quality of life, functionality, and community reengagement [7, 9, 26, 27]. In previous studies, cut-off point at 2.5 and above on the mean item score of the SR subscale was used to define moderate to high (referred to as “high”) stigma resistance and less than 2.5 for low stigma resistance [9, 28]. Despite its clinical importance and applicability, the SR of people with BD is not still well addressed in clinical settings and community affairs. In some western and Asian nations, there are few studies reporting the level and predictors of SR among people with BD [7–9]. For example, a study conducted at 13 European countries among people with BD or depression reported that the level of SR was 59.7% [7]. An Iranian study reported that the level of SR was 57.49% [29]. Studies conducted in Turkey and northern India also showed that the level of SR was found to be 62.2% and 28.6%, respectively [30]. Some studies have shown that the presence of traditional and supernatural beliefs about the cause of mental illness is related with stigmatizing attitudes and discrimination. For example, a study in Nigeria stated that subjects who believed in demonic possession as the cause of their mental illness were more likely to have internalized stigma and low SR ([31].

SR is the missed opportunity in African countries, particularly in Ethiopia. As per the authors' knowledge, there is only one study reporting the magnitude of SR among patients with mental illness in Ethiopia. However, it was limited to individuals with schizophrenia [28]. Therefore, this study is aimed at assessing the prevalence of SR and its predictors of people with bipolar disorders attending the outpatient department at AMSH. We hypothesized that low self-esteem, high internalized stigma, and unemployment were associated with low stigma resistance. The findings of this research work may have valuable clinical and policy implications by showing the level of stigma resistance and suggesting directions to design applicable strategies focusing on the promotion of stigma resistance and coping mechanisms of people with bipolar disorders. Furthermore, this study will be used as baseline information for further research works and community service programs.

## 2. Methods

### 2.1. Study Design and Period

This was an institutional-based cross-sectional study conducted from May 8^th^, 2016, to June 14^th^, 2016.

### 2.2. Study Setting

The current study was conducted at an outpatient department (OPD) of AMSH which is found in the capital city of Ethiopia, Addis Ababa. AMSH is the first hospital that started mental health service in Ethiopia since its establishment in 1930. The hospital provides services for people with psychiatric, neurological, substance use, and psychosocial problems in both outpatient and inpatient cares. Currently, the hospital has 18 OPDs serving for about 8,750 individuals with different mental health problems each month. The hospital has also 259 beds for inpatient service of people with severe mental illness, substance use, and emergency mental health conditions.

### 2.3. Source and Study Population

All bipolar patients who had a follow-up at the outpatient department of AMSH were the source population. Those who came for the follow-up during the data collection period were considered as the study population.

### 2.4. Inclusion and Exclusion Criteria

Clients with a confirmed clinical diagnosis of BD and age above or equal to 18 years during the data collection period were considered as eligible candidates for participation, whereas those who were on the acute episode and unable to speak or hear were excluded from the study.

### 2.5. Study Variables

The dependent variable was stigma resistance. Independent variables included sociodemographic factors (age, sex, ethnicity, religion, residence, marital status, educational status, and employment status), clinical factors (duration of illness, type of episode, duration of treatment, and number of hospitalization), and psychosocial factors (social support and self-esteem).

### 2.6. Sample Size Determination and Sampling Procedure

We used a single population proportion formula to estimate the minimum numbers of samples required for this study. Assumptions made for the sample size calculation were 50% prevalence of stigma resistance, 0.5*P*, 1.96*Z* (standard normal distribution), 95% CI, *α* = 0.05, and a 10% nonresponse rate. Accordingly, a sample was found to be 423.

Before the data collection, the total number of bipolar patients who visited the hospital in 2015 was identified from patients' records. Then, the average number of bipolar patients expected to visit the hospital during the data collection period was estimated to be 926. Based on this, a sampling interval (*K*) was determined by diving the total number of individuals with bipolar disorder expected to have a follow-up visit at the time of the data collection to the calculated sample size (*K* = 926/423 ≈ 2). Finally, eligible individuals were interviewed for every 2 intervals based on the order of their clinical evaluation at the OPD.

### 2.7. Data Collection Procedures

Data were collected via face-to-face interviews using a pretested semistructured questionnaire consisting of sociodemographic factors, clinical characteristics, Oslo-3 Social Support Scale, Rosenberg Self-Esteem Scale, and internalized stigma of mental illness questionnaires.

All contents of the questionnaire were first prepared in English and translated to Amharic (the official language of the country) by Amharic-speaking linguist. Then, back translation to English was also done by mental health specialists to check its consistency. Prior to the actual data collection, the Amharic version of the questionnaire was pretested among 21 individuals with bipolar disorder who have never been included in the actual data collection. Based on the pretest result, a minor modification was done regarding the contents of the questionnaire, and expressions of some statements were rephrased to be easily understandable by the study participants.

Six data collectors (BSc psychiatric nurses) and two supervisors (MSc level mental health professionals) participated in the data collection after attending training regarding the content of the questionnaire and data collection procedures. Data collectors evaluated the eligibility of individuals and requested written consent after delivering a brief explanation regarding the objectives of the study. The interview was conducted at a separate private room prepared for this purpose around the waiting hall of AMSH.

### 2.8. Instruments

The outcome variable (stigma resistance) was evaluated using the stigma resistance subscale of the Internalized Stigma of Mental Illness (ISMI) scale [25]. The ISMI has 29-item questions with four possible response options. These 29 questions have been grouped into five subscales reflecting stigma resistance, alienation, stereotype endorsement, perceived discrimination, and social withdrawal. In this study, we used the stigma resistance subscale with five-item Likert scale questions (1 = strongly disagree, 2 = disagree, 3 = agree, and 4 = strongly agree) measuring a person's ability to resist or be unaffected by internalized stigma.

Each of the five questions of the stigma resistance scale has four response options scored with a reverse direction yielding a sum score ranges from 1 to 4 points. In the current study, Cronbach's alpha for stigma resistance was 0.632. A cut-off point at 2.5 and above on the mean item score of the stigma resistance subscale was applied to define moderate to high (referred to as “high”) stigma resistance and less than 2.5 for low stigma resistance. This cut-off point has been applied in previous studies on stigma resistance [9, 28, 32].

The social support level of the study participants was assessed by using the Oslo-3 Social Support Scale (OSSS-3). The tool has three-item questions collectively used to measure the accessibility of support patients will receive from their family, friends, and neighbors if needed [33]. Oslo-3 Social Support Scale is a validated tool to be used to measure social support level of individuals at clinical settings and community level. The tool has been used in different clinical- and community-based studies of African countries including Ethiopia. It has a total sum score ranging from 3 to 14 points. Based on the total sum score, the level of social support was categorized as poor, moderate, and strong social support with sum scores of 3-8, 9-11, and 12-14, respectively.

The self-esteem of individuals with bipolar disorders was evaluated using the Rosenberg Self-Esteem Scale. The tool contains 10-item questions with four possible response options (from strongly agree (1) through strongly disagree (4) for each. Based on the sum score, the level of self-esteem of the study participants was categorized as low self-esteem (a sum score of <15) and high self-esteem (a sum score of ≥15) [34].

### 2.9. Data Processing and Analysis

The collected data were entered into EPI Info version 7 and exported to SPSS Windows version 20 program for analysis. Descriptive statistics (frequencies, tables, percentages, means, and standard deviation) were used to explain the sociodemographic characteristics, clinical variables, and level of SR of the study participants. Bivariable and multivariable analyses were applied to identify factors associated with SR. Accordingly, variables with *P* values of less than 0.2 in the bivariable analysis were considered as candidates for further multivariable analysis to control possible cofounders. In the multivariable model, variables with *P* values of less than 0.05 were considered as statistical predictors of SR. The odds ratio with a 95% confidence interval was used to measure the strength of the association.

### 2.10. Ethical Approval and Consent to Participation

This study was ethically approved by the Institutional Review Board (IRB) of the University of Gondar, and a supportive letter was obtained from Amanuel Mental Specialized Hospital. A written consent of participation was secured from each participant after delivering a brief explanation regarding the purpose and objectives of the study. Respondents were informed as they have full freedom to refuse and/or discontinue their participation at any time they want. Data collectors also disclosed that there will not any harm imposed towards respondents due to their willingness and/or refusal to participate. During the data collection, personal identifiers like name and phone numbers of respondents were not recorded. The collected data were kept confidential and used only for the purpose of the study.

## 3. Results

### 3.1. Sociodemographic Characteristics

From a total of 423 individuals invited to participate in this study, 418 participants completed the interview with a response rate of 98.81%. The mean (±SD) age of respondents was 34.29 (±10.36) years. Among respondents, 39.2% were within the age range of 25-34 years and more than half (53.3%) were not married. About 51.5% of the participants were males and more than half (55.0%) of them were orthodox in religion. Thirty-six percent of the study subjects were Amhara by their ethnicity and the majority (63.2%) of them were employed. Nearly one-third (33.5%) of bipolar patients attend secondary school education and 74.4% of them were from an urban area (Table 1).

### 3.2. Clinical and Psychosocial Characteristics

Regarding the clinical characteristics of the participants, nearly one-third (61.0%) of the participants developed BD before the age of 25 years and 37.6% of the reported that their illness had more than 10 years of duration. More than half (52.6%) of individuals with BD had attended an outpatient treatment follow-up for less than six years. The largest proportion (71.5%) of the study subjects had no family history of mental illness. Nearly seventy percent (68.2%) of the participants had high self-esteem and 42.1% of them reported poor social support (Table 2).

### 3.3. The Prevalence of Stigma Resistance

More than half (56.9%) of people with BD attending outpatient follow-up visits at AMSH scored below the mean score of a stigma resistance subscale of ISMIS with 95%CI = 51.9‐61.6%. Better SR level was observed among married individuals, individuals with good self-esteem, strong social support level, and better educational status.

### 3.4. Factors Associated with Stigma Resistance

To determine the association of independent variables with stigma resistance, bivariable and multivariable binary logistic regression analyses were performed. In the bivariable analysis, factors including employment status of the participants, marital status, educational level, current episode, number of previous episodes, internalized stigma, self-esteem, and social support were significantly associated with stigma resistance at a *P* value less than 0.2. These factors were entered into the multivariable logistic regression model to control confounding effects. However, in the multivariable analysis, being unemployed (AOR = 1.65; 95%CI = 1.35‐3.87), having a high level of internalized stigma (AOR = 3.04; 95%CI = 1.83‐5.05), and low self-esteem (AOR = 2.13; 95%CI = 1.72‐6.76) were found as statistically significant predictors of stigma resistance at *P* value less than or equal to 0.05 (Table 3).

## 4. Discussion

The finding of this study showed that the stigma resistance level of individuals with bipolar disorder at AMSH is low. More than half, of people with bipolar disorder attending outpatient follow-up visits at AMSH, scored below the mean score of stigma resistance subscale of ISMI. This figure is in line with studies done across 13 countries in Europe (59.7%) [7] and Iran (57.49%) [29]. However, the finding of the current study was higher as compared to a study conducted in northern India which reported that 28.6% of bipolar patients had low stigma resistance [35]. The possible explanation for the discrepancy of such findings might be due to the differences in population characteristics and sample sizes (the Indian study used 185 patients with bipolar disorder). The collectivism culture of the Ethiopia population might play a role to resist stigma as a result of a better social support system and community reengagement. On the other hand, a study conducted in Turkey (62.2%) [30] showed a better stigma resistance of people with bipolar disorder as compared to the finding of our study. Differences in the cultural and sociodemographic characteristics of the study participants might play a role in the difference of these findings. For example, low educational status and unemployment status of individuals in Ethiopia may be possible reasons for the lower stigma resistance ability of the study participants. High educational status such as university education may contribute to having high stigma resistance by helping the people not to apply the devaluing judgments to themselves [36, 37].

The second objective of this study was to identify predictors of SR among individuals with bipolar disorder. Participants who were currently unemployed were 1.65 times more likely to have low SR as compared to those who were employed. This finding is in line with other studies [38, 39]. The possible explanation for this discrepancy might be due to the fact that employed individuals often have better confidence and become mentally satisfied as a result of their reliance. This suggests a need to develop strategies to have high stigma resistance by enhancing the employment status of individuals with mental health problems.

Bipolar patients who had a higher internalized stigma were more than three times more likely to have lower stigma resistance as compared to their counterparts. This finding is supported by previous studies that reported higher stigma resistance was related to lesser internalized stigma [32, 40]. This might possibly be explained by the fact that patients who had high stigma resistance may not apply negative stereotypes such as devaluation, shame, secrecy, and withdrawal [9, 25, 41]. The collaboration between health sectors and community support group is needed to minimize internalized stigma and improve the stigma resistance capacity of bipolar patients.

Finally, the study participants who had low self-esteem were 2.13 (AOR = 2.13; 95%CI = 4.72‐8.76) times more likely to have low SR. A study in northern India also stated that there is a positive correlation between stigma resistance and self-esteem in patients with mental illness [9]. Low self-esteem reduces the patient's capacity of stigma resistance by applying negative stereotype attitudes about themselves [42]. Therefore, promotion and strengthening of positive self-esteem can be considered as a strategy to improve the SR capacity of people with BD.

### 4.1. Limitations of the Study

This study may have limitations. First, the study was conducted among individuals with bipolar disorder having outpatient follow-up visit and might not be representative of individuals with mental health problems other than bipolar disorder. Second, as far as the study was conducted at a hospital level, bipolar patients who did not start a treatment have never been addressed. Third, the cross-sectional nature of the study design might not show the cause and effect relationships of study variables. Fourth, the data collection method was a face-to-face interview which might led individuals to respond in socially acceptable ways (social desirability and recall bias). Finally, since the instrument we have used to assess SR is not validated in Amharic language (local language of our study area), it might under- or overestimate the SR.

### 4.2. Conclusion and Recommendation

The finding of this study showed that more than half of individuals with bipolar disorder had low stigma resistance. Stigma reduction programs focusing on improving the self-esteem and minimizing the internalized stigma were better to be implemented by stakeholders to increase the stigma resistance of patients with bipolar disorder. Moreover, mental health information dissemination regarding the community support and reengagement of people with bipolar disorder is highly recommended. Validitating the stigma resistance subscale of the internalized stigma of mental illness in local language is recommended for researchers.

## Figures and Tables

**Table 1:** Sociodemographic characteristics of people with bipolar disorder attending the outpatient department at Amanuel Mental Specialized Hospital, Addis Ababa, Ethiopia, 2016 (*n* = 418).

Variables	Categories	Frequency	Percentage
Age in years	18-24	66	15.8
	25-34	164	39.2
	35-44	120	28.7
	≥44	68	16.3

Sex	Male	216	51.5
	Female	202	48.3

Religion	Orthodox	230	55.0
	Muslim	94	22.5
	Protestant	82	19.6
	Others^∗^	12	2.9

Marital status	Single	223	53.3
	Divorced/widowed	65	15.6
	Married	130	31.1

Ethnicity	Amhara	151	36.1
	Oromo	120	28.7
	Guragie	75	17.9
	Others^∗∗^	72	17.2

Educational status	Unable to read and write	62	14.8
	Primary	103	24.6
	Secondary	140	33.5
	College and above	113	27.0

Residency	Rural	107	25.6
	Urban	311	74.4

Employment status	Employed	264	63.2
	Unemployed	154	36.8

^∗^Catholic and Adventists. ^∗∗^Tigre, Silte, Gamo, Woleni, and Wolaiyta.

**Table 2:** Clinical and psychosocial factors of people with bipolar disorder attending the outpatient department at Amanuel Mental Specialized Hospital, Addis Ababa, Ethiopia, 2016 (*n* = 418).

Variables	Categories	Frequency	Percentage
Age at onset of illness	≤25 years	255	61
	>25 years	163	39

Duration of illness	Less than 5 years	147	35.2
	5-10 years	114	27.3
	>10 years	157	37.6

Treatment duration	≤6 years	220	52.6
	>6 years	198	47.4

Number of episodes	<2	167	40
	≥2	251	60

Hospitalization history	Yes	218	52.2
	No	200	47.8

Type of current episode	Manic	310	74.2
	Depressive	108	25.8

Practice of traditional treatment	Yes	190	45.5
	No	228	54.5

Family history of mental illness	Yes	119	28.5
	No	299	71.5

Previous suicidal attempt	Yes	144	34.4
	No	274	65.6

Medication discontinuation	Yes	233	55.7
	No	185	44.3

Social support level	Poor	176	42.1
	Moderate	148	35.4
	Strong	94	22.5

Self-esteem	Low self-esteem	133	31.8
	High self-esteem	285	68.2

**Table 3:** Bivariable and multivariable analyses of stigma resistance among people with bipolar disorder attending the outpatient department at Amanuel Mental Specialized Hospital, Addis Ababa, Ethiopia, 2016 (*n* = 418).

Variables		Stigma resistance		COR (95% CI)	AOR (95% CI)
		Good	Poor		
Employment status	Employed^a^	101	163	1.00	
	Unemployed	79	75	1.69 (1.14-2.54)	**1.65 (1.35-3.87)**

Marital status	Single	97	126	1.61 (0.90-2.89)	1.45 (0.88-2.39)
	Divorced/widowed	62	68	1.91 (1.02-3.56)	0.57 (0.29-1.08)
	Married^a^	21	44	1.00	1.00

Educational status	Unable to read and write	34	28	1.90 (1.102-3.56)	1.54 (0.76-3.12)
	Primary	42	61	1.02 (0.63-1.86)	1.02 (0.57-1.84)
	Secondary	60	80	1.18 (0.71-1.95)	1.09 (0.63-1.88)
	College and above^a^	44	69	1.00	1.00

Current episode	Manic^a^	141	169	1.00	1.00
	Depressive	39	69	0.68 (0.43-1.06)	1.24 (0.74-2.06)

Number of episodes	<2 years^a^	64	103	1.00	1.00
	≥2 years	116	135	0.72 (0.48-1.08)	0.74 (0.47-1.17)

Internalized stigma	Yes	112	202	2.94 (0.18-0.48)	**3.04 (1.83-5.05)**
	No^a^	68	36	1.00	1.00

Self-esteem	Low	63	117	1.29 (0.85-1.96)	**2.13 (1.72-6.76)**
	High^a^	70	168	1.00	1.00

Social support level	Poor	82	94	1.77 (1.05-2.98)	1.40 (0.02-3.20)
	Moderate	67	81	1.68 (0.98-2.88)	1.75 (0.98-3.09)
	Strong^a^	31	63	1.00	1.00

^a^Reference variable. OR: crude odds ratio; AOR: adjusted odds ratio.

## Data Availability

The datasets generated and/or analyzed during the current study are not publicly available due to some privacy reasons, but part of the row datasets will be available from the corresponding author on reasonable request.

## Abbreviations

AMSH: Amanuel Mental Specialized Hospital
AOR: Adjusted odds ratio
BD: Bipolar disorder
BSc: Bachelor of Science
CI: Confidence interval
COR: Crude odds ratio
ISMI: Internalized stigma of mental illness
MSc: Masters of Science
OPD: Outpatient departments
OSSS-3: Oslo-3 Social Support Scale
SD: Standard deviation
SR: Stigma resistance
WHO: World Health Organization.
